# Pilot Test of Diabetes Screening and Referral to Primary Care Within an Alaska Native Dental Clinic: Protocol for a Feasibility Study

**DOI:** 10.2196/75619

**Published:** 2026-03-04

**Authors:** Kathryn R Koller, Julie A Beans, Diane K King, Vanessa Y Hiratsuka, Gretchen M Day, Shiela M Strauss, Barbara J Stillwater

**Affiliations:** 1 Research Services, Alaska Native Tribal Health Consortium, Anchorage, AK, United States; 2 Research Department, Southcentral Foundation, 4085 Tudor Centre Drive, Research Department, Anchorage, AK, 99508, United States, 1 907-729-4333; 3 Center for Behavioral Health Research Services, University of Alaska Anchorage, Anchorage, United States; 4 Rory Meyers College of Nursing, New York University, New York, NY, United States

**Keywords:** diabetes mellitus type 2, Alaska Native, American Indian, prevention, dental

## Abstract

**Background:**

Recognizing the bidirectional relationship between oral health and diabetes mellitus (DM) and integrating DM screening and referrals to primary care into routine dental hygiene visits has strong potential to expand access to diabetes prevention and early detection services.

**Objective:**

This paper describes methods used in an academic-Tribal partnership to co-design, coimplement, and pilot-test a DM screening and referral process within a Tribal health care setting.

**Methods:**

The project uses implementation science frameworks and applies a 4-step iterative, participatory planning approach. Project implementation steps include (1) conducting key informant interviews with dental and primary care staff and administrators and surveying dental care recipients to assess the context for implementation and receptivity toward the proposed innovation; (2) forming and engaging a multidisciplinary planning team comprising dental, primary care, informatics, and care improvement personnel, care recipients, and researchers; (3) applying survey and interview findings and internal knowledge of clinic procedures and processes to develop a feasible dental DM screening service model to pilot test with iterative data collection; and (4) refining and disseminating the innovation.

**Results:**

Key informant interviews and patient surveys were completed in September 2023. The planning team then developed the pilot process based on these findings in combination with lived experience. Study enrollment for the pilot began in April 2025 and was completed in July 2025. Data analysis is underway.

**Conclusions:**

Offering DM screening during dental appointments aligns dental and primary care services to prevent or delay DM onset. This paradigm shift has the potential to facilitate communication between dental and primary care providers, improve the coordination of care across dental and medical services, capitalize on opportunities to reach individuals in need of DM screening while they are accessing other services, and expand health system capacity to identify individuals at risk and offer them appropriate DM services.

## Introduction

Across the lifespan, oral health both influences and is influenced by general health, and one of the most common health conditions encountered in dental clinics that reflects this relationship is type 2 diabetes mellitus (DM) [1-4]. Elevated blood sugar levels in DM adversely affect oral health, manifesting as oral diseases, including periodontal disease. In the United States, an Indian Health Service brief posted in 2022 reported that while oral health has improved among Alaska Native and American Indian (ANAI) Peoples, this population is more than twice as likely to have periodontal disease as the general US population [5]. DM prevalence among ANAI adults is approximately twice that of US White adults [6]. Clinical interventions to improve access to DM or pre-DM screening through novel venues among this population are underreported in the literature, despite surveillance data demonstrating the importance of oral health for persons diagnosed with diabetes and the potential for ANAI dental settings to serve as a screening and referral resource [7].

DM is the fifth leading cause of ANAI mortality for both adult men and women, in all age groups [8]. The medical needs of ANAI people with DM are complex because DM in this aggregated population is characterized by early onset and high rates of comorbidities (eg, heart disease, kidney failure, and lower limb amputation) [9]. A disease once rare among Alaska Native people specifically, DM now registers prominently among chronic diseases impacting Alaska Native people; its increased prevalence is attributed to the diet and lifestyle impacts of colonialism [10,11]. Findings from the Alaska Education and Research Towards Health baseline cohort study document the prevalence of modifiable risk factors contributing to DM progression (ie, tobacco use [12], obesity [12,13], large waist circumference [13], gestational diabetes [14], and physical inactivity [15]) among ANAI study participants between 2004 and 2006. A follow-up study conducted with a subset of urban Education and Research Towards Health participants during 2015 to 2017 [16] reported DM incidence of 16.5 per 1000 person-years and a pre-DM incidence of 77.6 per 1000 person-years [17]. DM prevalence (12% at baseline in 2004‐2006) had increased to 25% and pre-DM prevalence (31% at baseline) had risen to 70% in 10 years, with mean participant age increasing from age 40 to 50 years [17].

The American Diabetes Association recommends DM screening for anyone overweight or obese if they present with additional risk factors [18]. Additional risk factors include (but are not limited to) physical inactivity, first-degree relative with DM, high-risk race or ethnicity (including ANAI people), smoking, hypertension, dyslipidemia, and blood sugar levels in the pre-DM range. For all adults without risk factors, the American Diabetes Association recommends screening beginning at age 35 years. Those with risk factors who do not routinely seek primary care are at the greatest risk.

Any oral infection with associated local and systemic inflammatory responses adversely affects blood glucose levels [4,19]. As DM is intricately interwoven as both a risk and a complicating factor in periodontal disease [1], dental hygiene appointments scheduled more frequently for patients with periodontal disease or those with same-day appointments for urgent dental care provide important opportunities to identify undiagnosed DM through screening in this higher risk group [20]. This study aims to develop, test, and evaluate a DM screening and referral service offered in a Tribal dental clinic.

## Methods

### Ethics Approvals

The Alaska Area Institutional Review Board (protocol 2017-02-004) reviewed and approved this study. Tribally owned and operated health care organizations, the Alaska Native Tribal Health Consortium (ANTHC) and Southcentral Foundation (SCF) research review committees reviewed and approved this study and this manuscript before journal submission [21]. The study is being conducted by ANTHC and SCF, and data collected are owned by SCF. Data access requests for data collected under this protocol will have to be submitted, reviewed, and approved by both ANTHC and SCF.

### Population and Setting

SCF is an Alaska Native–owned, nonprofit health care organization operating under the Tribal authority of Cook Inlet Region, Incorporated. SCF provides dental and primary health care services for more than 70,000 ANAI people [22,23]. ANAI persons with periodontal disease are at increased risk for DM [19,24]. Those without regular primary care visits may not realize their risk for DM in the absence of discomfort or other physical symptoms, as is the case with early DM or pre-DM [19,25-27]. While studies in other population sectors have explored DM screening in dental clinics with varying degrees of success [28-35], none were conducted in a Tribal health system offering primary and dental services with shared electronic health records (EHRs).

SCF and ANTHC have research departments nested within the health care systems. These research departments conduct health research that is driven by and in service of ANAI people by ANAI people. This study’s project team comprised ANAI investigators as well as non-ANAI investigators. The study team works to ensure data interpretations include ANAI perspectives and experiences, which is why we also receive community-level review of data interpretation from both Tribal organizations before dissemination.

### Preparatory Work

SCF dental providers see more than 25,000 ANAI people from the Southcentral Alaska region annually. These findings have recently been corroborated (Koller KR, Hiratsuka VY, Day G, unpublished data, September 2017) by this research team between November 1, 2015, and April 30, 2017, showed at that time approximately 16% (n=4180) of dental clinic visits were coded as “same-day” (emergent or urgent) visits. Another 6% (n=1493) were coded by dental hygienists for periodontal disease care. Among 6795 unique individuals who did not have diagnosed DM, more than 76% (n=1722) of men and 55% (n=1872) of women seen on a same-day appointment basis had not been screened for DM during that 18-month period, despite the presence of DM risk factors. Specifically, nearly 54% were aged 45 years or older. Among those with scheduled hygiene appointments, 61% of men and 47% of women were unscreened for DM, and 42% were older than 45 years. Among ANAI individuals younger than 45 years, prevalent tobacco use (51% of same-day and 41% of hygiene appointment attendees) and obesity (approximately 40% in both clinic settings) indicate increased DM risk. These data support DM screening in the dental clinic setting for those being seen for same-day urgent care or for periodontal hygiene, with subsequent referral of those identified as at increased DM risk to their primary care provider. As early diagnosis and intervention during pre-DM can prevent DM onset, the service we propose could substantially reduce future DM incidence.

### Study Framework

This study uses community-based participatory research principles in the context of Tribal sovereignty [36,37]. Our study builds on previous research exploring the potential to conduct DM screening and referral during dental appointments, particularly for patients who do not regularly access primary care [20,28,30,35,38,39], and adapts these evidence-informed recommendations for the Tribal health setting.

**Figure 1.**
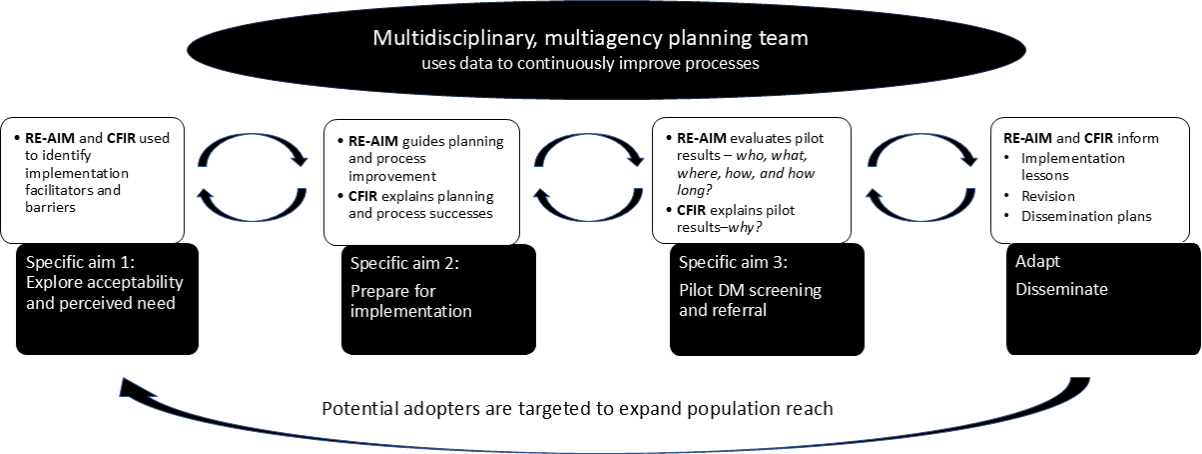
Use of dissemination and implementation frameworks to promote external validity. CFIR: Consolidated Framework for Implementation Research; DM: diabetes mellitus; RE-AIM: reach, effectiveness, adoption, implementation, and maintenance.

We adapted the Centers for Disease Control and Prevention’s *Replicating Effective Programs* Implementation Science framework, which recommends four steps to guide successful implementation (Figure 1) [40,41]: (1) explore the acceptability and perceived need of the intervention, (2) prepare for preimplementation, (3) pilot-test and refine processes based on monitoring data and feedback, and (4) adapt and disseminate.

We will use the reach, effectiveness, adoption, implementation, and maintenance (RE-AIM) framework [42] to inform implementation strategies, use relevant process evaluation metrics, and evaluate implementation success [43]. We also included constructs from the Consolidated Framework for Implementation Research (CFIR) [44] to explore factors that explain RE-AIM outcomes (Figure 1) and provide an iterative systematic approach to design, pilot test, refine, and evaluate the screening and referral service [45,46].

### Planning Team

Health system user input is critical in designing efficient, effective services that are acceptable to all involved, resulting in its optimal use [47]. Thus, together with an experienced study team serving as practice change facilitators, we will assemble a multidisciplinary, multiagency planning team that includes ANAI individuals who may both receive and provide dental and primary care at SCF, along with SCF internal process improvement staff representing both dental and primary care services. Using dissemination and implementation science frameworks, the planning team will collect formative information from groups involved with the health system to design implementation procedures and pilot test them.

Health system representatives are individuals with authority to make system-level changes (eg, to the EHR, training requirements, and equipment) and staff involved in service delivery (eg, primary care providers, dental providers, and health educators), quality improvement, data services, health information technology, administration, and Tribal leadership. Staff will include both ANAI and non-ANAI individuals. Volunteers from these areas will participate in the study planning team. The team will decide meeting frequency and communication methods. The planning team’s meetings will focus on reviewing and providing input on activities across the entire study, including hands-on development of implementation, iterative process data collection and review protocols, pilot test planning, interpretation of preliminary analyses, and review and dissemination of findings.

### Study Design and Overview

We will use a within-site, postdesign, phased approach [41] to study the implementation of an evidence-based DM preventive service delivered in this novel dental clinic setting [48]. In the first 2 years, we will use a convergent parallel mixed methods design [49] to analyze health system barriers and facilitators to implementing the DM screening and referral service and codevelop a dental clinic DM screening and referral service with dental and primary care clinic patients and staff (Table 1). In years 3 and 4, we will pilot-test the service, using RE-AIM metrics to evaluate reach, implementation fidelity, and the potential for setting-level maintenance. We will thematically code planning team meeting minutes, surveys, and interview transcripts using CFIR constructs to describe observed determinants that facilitated or were detrimental to reaching patients and implementing the intervention as planned.

**Table 1.** Research activities timeline.

	Year 1				Year 2				Year 3				Year 4			
	Q1^a^	Q2^b^	Q3^c^	Q4^d^	Q1	Q2	Q3	Q4	Q1	Q2	Q3	Q4	Q1	Q2	Q3	Q4
Planning team meetings	✓	✓	✓	✓	✓	✓	✓	✓	✓	✓	✓	✓	✓	✓	✓	✓
IRB^e^ and Tribal approvals	✓	✓														
Dental clinic and primary care staff Interviews—data collection and analysis	✓	✓	✓	✓	✓											
Dental patient surveys—data collection and analysis		✓	✓	✓	✓											
Develop intervention clinic workflows						✓	✓	✓								
Train dental staff								✓	✓							
Enroll 60 dental patients										✓	✓	✓				
Electronic health record review at 1, 3, and 6 weeks postenrollment											✓	✓	✓			
CFIR^f^ and REAIM^g^ evaluation													✓	✓		
Summarize findings and present to Tribal leadership, internal departments, and community members														✓	✓	✓

a Q1: quarter 1.

b Q2: quarter 2.

c Q3: quarter 3.

d Q4: quarter 4.

e IRB: institutional review board.

f CFIR: Consolidated Framework for Implementation Research.

g REAIM: Reach, Effectiveness, Adoption, Implementation, and Maintenance.

### Dental Clinic and Primary Care Staff Interviews

Staff interviews will be used to understand factors relevant to referral service adoption, implementation, and maintenance. The guide is embellished with questions from the CFIR interview guide [50], which aim to explore the *outer* and *inner setting*, *individual characteristics,* and *process* constructs (Textbox 1). Such constructs include health care professional beliefs that the service is needed; will achieve the desired outcomes; and will fit with existing norms, culture, work processes, and practices.

Textbox 1. **Interview guide.**

Introduction

Diabetes is a significant health concern and an SCF (Southcentral Foundation) health priority. Our research team is exploring how to pilot diabetes screening for adults in the dental setting. The presence of moderate or severe periodontal disease is a significant risk factor for and a complication of diabetes. Due to this relationship, one method we are exploring is to screen adults for diabetes while they are at the dental clinic using a quick finger-stick blood test. We are also interested in screening adults in the walk-in dental clinic, as some of these individuals do not receive regular dental or primary care and may be at risk for diabetes.

a. Does the SCF dental clinics currently screen for chronic disease? Describe how that happens. Probes: can you describe the protocol in place for referral to primary care? If it does not happen, describe what might need to change to make this feasible. When in a dental visit does chronic disease screening and/or referral occur? What staff are involved? How are other chronic disease screening processes coded or billed? What issues do you foresee with respect to billing or coding diabetes screening in the dental clinic?

b. Does SCF Dental support individuals with diabetes currently? Probe: if yes, can you describe the support provided by SCF Dental for individuals with diabetes? Does SCF Dental support individuals with prediabetes currently? If so, can you describe how SCF Dental supports individuals with prediabetes?

c. Is the relationship between oral health and diabetes recognized by dental professionals? Probe: would diabetes screening and referral during a routine dental clinic visit fill an existing health need? Describe how.

d. Would adding diabetes screening and referral to primary care be feasible for SCF dental clinic to do now? Why or why not? Probe: what competing priorities do you see for implementing diabetes screening and referral in the dental clinic?

e. Whose buy-in will be necessary to successfully implement diabetes screening and referral in SCF dental clinics? Probe: who are current champions? Do you have suggestions on how we can best minimize resistance and/or disruptions to implementing diabetes screening and referral in SCF dental clinics?

f. What changes might be necessary to implement before introducing a new screening protocol (eg, documentation, billing, policies and procedures, and reporting)? Probe: whose work will be most impacted by adding diabetes screening and referral to primary care? How will it be impacted? Who in the dental clinic should or could screen for diabetes?

g. Screening encompasses several steps, including asking the customer-owner (SCF term for patients) about their diabetes status using a questionnaire, reviewing the questionnaire to determine whether to do a diabetes screening blood test, collecting and analyzing the blood sample, and then providing a referral and documenting the results in the patient record. Could or should different people be involved in these steps?* (notes which questions can be skipped if running short on time) Probe: who could or should do what? Can you foresee potential issues getting buy-in or acceptance of these duties? What skills and training will likely be needed? How do you train people now when introducing a new skill or procedure? How are new staff typically trained on other types of screening done in your practice?

h. Given that screening results may identify those currently at risk for diabetes as well as those with probable diabetes, who in your practice would be best positioned to relay the results, provide education, and coordinate next steps? Probe: can you foresee potential issues getting buy-in or acceptance of this role? Describe.*

i. What quality indicators do you currently use to monitor and make improvements to service delivery as well as customer-owner outcomes (eg, chart review, dashboard reports, staff, and customer-owner feedback)? What sorts of data would be helpful to monitor the success of a diabetes screening program in dental clinics?

j. Describe what you see as the main challenges to implementing diabetes screening as a part of routine dental care? Probe: are there system changes needed, such as health record documentation and prompts? Are there logistical barriers, such as staffing, scheduling, and workflow that would need to be addressed? Are there situations where we should not screen for diabetes in the dental clinic?

k. What types of mitigation practices were in place during the COVID-19 pandemic? Probe: are these practices still in place? Will these practices change how diabetes screening is managed in the dental clinic?*

l. Are there any other considerations that should be kept in mind or addressed before, or as part of, the diabetes screening pilot project?

Key informant participants will represent health system staff and be determined by the planning team, including representatives from dental and primary care center administration leadership and clinical providers at all levels. We will conduct at least 15 in-depth interviews to reach data saturation on core constructs. Key informant participants will receive a promotional item following interview completion. Experienced study team members will use ATLAS.ti (version 23.3.2.28729 for Windows) for qualitative analysis. Interviews will be coded using template analysis [51,52], with 2 coders reviewing each transcript to ensure intercoder reliability. Discrepancies will be discussed and resolved by consensus. Guided by CFIR domains and discussion with the planning team, template analysis will be used to identify barriers and facilitators.

### Preimplementation Preparation

To plan for implementation, the planning team will engage in audio-recorded reflective planning sessions in which members will discuss the planning process, completed milestones, new or unresolved implementation barriers, and needed refinements. During these sessions, patient surveys and key informant interview data will be reviewed to identify barriers, facilitators, and perceptions (eg, patient and staff beliefs about the relative importance, value, and need for the service, and feasibility). The planning team will also codevelop intervention delivery protocols designed to be integrated, feasible, sustainable, and culturally relevant for ANAI patients. We will use a systems-level design approach to maximize patient reach, effectiveness, and buy-in from clinicians (adoption). The team will meet monthly via videoconference and quarterly for in-person sessions.

Using ATLAS.ti (version 23.3.2.28729 for Windows), experienced members of the investigative team will code planning team meeting minutes, reflective session notes, and recorded meeting transcripts for RE-AIM domains and CFIR themes. These investigative team members will assess the extent to which reach, effectiveness, staff buy-in and training (adopters), implementation logistics, service quality, and long-term maintenance are addressed and will ensure that mechanisms for periodic refinement of implementation plans are in place. Analysis includes planning team composition characteristics, member participation rates, and other evidence of CFIR readiness for implementation and process determinants. Process determinants include leadership engagement (eg, acceptance and completion of assignments and responsiveness to requests for help), allocation of resources (eg, time for planning and training and funds for infrastructure changes), internal implementation leaders, and communication with stakeholders. Experienced members of the investigative team will provide the qualitative results to the planning team, which will further refine plans for obtaining clinic-level feedback and suggestions and monitor quality throughout the pilot study.

### Pilot DM Screening and Referral

Once the planning team finalizes screening and referral protocols, the investigative team will train and certify clinicians or staff in all protocols. The planning team will make a final determination on which dental providers and clinics will be used. Inclusion criteria will be SCF dental patients aged 18 years or older being seen on a walk-in basis or for a prescheduled cleaning by a dental hygienist, ANAI heritage, at least 1 self-reported additional DM risk factor (eg, first-degree relative with DM, smoking, hypertension, dyslipidemia, and older than 35 years), and not currently managed for DM.

Interested individuals will be screened for eligibility by the investigative team in the dental clinic lobby. A study team member will go through the informed consent form with the potential participant and provide time for the individual to ask questions. Participants who provide informed consent will be assigned a unique anonymous study code stored in REDCap (Research Electronic Data Capture (REDCap; Vanderbilt University).. We anticipate demographic, contact, and basic DM risk factor information collected via an iPad survey to be linked in REDCap to each participant’s study code. Clinic staff may measure height and weight, perform finger-stick glycated hemoglobin testing using planning team–selected point of care analyzers (eg, A1cNow, Affinion, or DCA Vantage), and initiate the screening and referral service for participants who provide informed consent.

The pilot will enroll 60 participants and will follow those referred to primary care through the dental screening and referral service to monitor process flow and follow-up and to iteratively refine the referral service. Adequate power to assess differences will not be an issue, as we will evaluate the screening and referral implementation rather than measure individual-level health outcomes. Upon signed informed consent, trained and experienced study staff will conduct EHR reviews at 1, 3, and 6 weeks postenrollment to monitor primary care follow-up. Pilot test participants will receeive a US $40 gift card at enrollment.As recommended, members of the investigative team will conduct regular practice facilitation calls with pilot testing clinics to iteratively identify challenges and successes, facilitate problem-solving, update the planning team, and refine protocols [53].

The primary outcome of this study is the assessment of the implementation process, resources required, and management needed for data collected during intervention implementation [54]. We will use descriptive statistics to describe the pilot study sample. We will collect implementation quality data using RE-AIM metrics and CFIR constructs, including EHR documentation of screening rates (reach), training rosters and evidence of leadership and clinician engagement (adoption), documentation of executing screening and referral services as planned (implementation), and planning team completion of a sustainability checklist of evidence-based indicators [47,55]. These indicators support continued postpilot test delivery of the service if demonstrated to be effective, including commitment of available resources (funding, training, and time) for implementation and ongoing operations (maintenance). We will conduct follow-up surveys with providers and pilot participants using REDCap [56] links to evaluate satisfaction with service delivery, suggestions for improvement, and system-level and patient effectiveness outcomes. Follow-up survey respondents will receive a US $15 gift card (pilot participants only) or a promotional item.

Using mixed methods, we will analyze the following RE-AIM data:

- Reach: number, percent, and representativeness of eligible pilot study patients who consent to screening in comparison to the overall population of eligible patients
- Effectiveness: success in engaging patients in testing and follow-up with a primary care provider, measured by the number and percent of referred patients completing a primary care visit where DM risk was addressed, as well as differences in completion rates by key demographic factors (ie, chi-square)
- Adoption: number, percent, and representativeness of invited providers and staff completing training
- Implementation: extent to which screening and referrals were conducted according to service protocols, number and percent of positive screens receiving a referral, number and percent of referred patients who complete a primary care appointment, and use of process data to adapt protocols
- Maintenance: results from a sustainability checklist developed by the planning team and team reflections on persistent barriers and intentions to adopt and disseminate the screening and referral service permanently

In the final step, evaluative data will be provided to the Tribal health care system leadership via the planning team to determine needed adaptations for dissemination beyond the pilot test.

## Results

Dental and primary care staff interviews and patient surveys began in March 2023 and were completed in September 2023. The planning team has met a total of 12 times and used data gathered from the interviews and surveys to supplement their lived experience in planning the pilot study process. The pilot study began enrollment in April 2025 and completed it in July 2025. Data analysis is underway.

## Discussion

### Anticipated Findings

There is strong evidence of the robust bidirectional connection between periodontal disease and DM [57] and the urgent need to reach ANAI adults with and without periodontal disease for early identification and intervention to prevent the onset of undiagnosed or uncontrolled DM [17]. Our proposed methods to study the development and implementation of an evidence-based DM screening and referral service, which expands the role of dental practices to include DM screening and promotes care coordination among dental and primary care providers, are timely and relevant to national goals to address DM [58].

The major strengths of this study are (1) focused attention on dental services likely to have a greater proportion of the ANAI population with increased risk for DM, thereby maximizing screening efforts; and (2) referral to and follow-up with primary care providers for ongoing treatment and monitoring. Results from this study will be applicable to integrated health care systems, Tribal health care beneficiaries, and health systems operating patient-centered medical and/or dental home models.

### Limitations

A limitation is that not all individuals at risk will be screened. However, successful implementation may pave the way for extending screening to other services in the future. In addition, our study of a single Tribal health care system will limit the generalizability of our findings, given the heterogeneity of dental and health care delivery across the United States.

### Conclusions

The lessons and strategies for implementing a service to reduce the burden of undiagnosed DM and multimorbidity complications will be disseminated throughout SCF dental services as well as among other Tribal health organizations and dental health aide therapist programs. They may then be adapted and applied in Tribal health settings and rural regions of the nation. This project will also set the stage for a large-scale, sufficiently powered hybrid effectiveness [59]—or pragmatic, pretest-posttest, multisetting—implementation trial with long-term follow-up. The goal of such a large trial will be to supply vital information about the most feasible implementation strategies and outcomes when delivering coordinated dental–primary care services to prevent DM.

## Abbreviations

ANAI: Alaska Native and American Indian
CFIR: Consolidated Framework for Implementation Research
DM: diabetes mellitus
EHR: electronic health record
RE-AIM: reach, effectiveness, adoption, implementation, and maintenance
REDCap: Research Electronic Data Capture
SCF: Southcentral Foundation
